# Recognition of adaptive stress response patterns in adolescents based on multiple hair biomarkers

**DOI:** 10.3389/fpsyg.2026.1720146

**Published:** 2026-04-13

**Authors:** Shurui Shen, Weiliang Wang, Shan Su, Sihan Liu, Hao Shen

**Affiliations:** School of Nursing, Xuzhou Medical University, Xuzhou, China

**Keywords:** adaptive calibration model, adolescents’, hair cortisol, HPA axis, stress

## Abstract

**Background:**

The Adaptive Calibration Model (ACM) explains how individuals’ stress response systems (SRS) develop different calibration patterns under environmental stress, subsequently shaping physiological responses and behavioral strategies during growth. However, research on adaptive stress response patterns has yielded inconsistent results across studies, warranting further investigation.

**Methods:**

This study used hair cortisol, cortisol/DHEA ratio (CD), and cortisol/cortisone ratio (CC) as biomarkers reflecting multidimensional functional indicators of Hypothalamic–Pituitary–Adrenal (HPA) axis adaptation. Latent profile analysis identified heterogeneous stress adaptation patterns in adolescents (*N* = 124, age range: 12–18). We compared differences in these patterns across sex and disadvantaged/non-disadvantaged groups, and examined their relationship with depressive symptoms.

**Results:**

A 4-profile solution was selected based on theoretical interpretability. The buffered profile was most prevalent (35.5%). Children from disadvantaged backgrounds, compared to non- disadvantaged children, were characterized by a significantly higher proportion of the vigilant profile. However, no significant differences in depression scores were found among the different stress adaptation patterns.

**Conclusion:**

This study provides the first validation of ACM-proposed stress adaptation patterns from multidimensional functional indicators of HPA axis functional perspective. Early disadvantaged environments appear to promote vigilant stress adaptation patterns. These findings provide valuable reference for future mechanism analysis and intervention development for stress-related conditions in adolescents.

## Introduction

Stress accompanies individuals throughout their entire life cycle, and the analysis of stress mechanisms has consistently been a topic of significant interest. Stress research has long focused on the negative impacts of stress on individuals, including psychological disorders such as anxiety and depression, as well as physiological problems involving the cardiovascular and immune systems. At the same time, an expanding body of research has shown that individuals do not always respond to stress in uniformly negative ways, highlighting adaptive and positive responses that complement the traditional deficit-focused perspective. Instead, they flexibly adjust their physiological and psychological states according to their own resources and environmental demands. This flexible adjustment mechanism is the “adaptive calibration” emphasized by the Adaptive Calibration Model (ACM) model (Del Giudice et al., 2011).

The ACM model explains individual differences in stress response system (SRS) functioning (Del Giudice et al., 2011). To promote adaptation, the SRS not only determines how to cope with stress “in the moment” but also determines what kind of adaptive strategies individuals maintain throughout their development (Del Giudice et al., 2011). Early experiences (such as family environment and social support) and environmental factors (resources, dangers, unpredictability) may cause individuals to experience different plasticity windows (Wagner et al., 2024), affecting the long-term adjustment and developmental trajectory of the SRS. Based on the heterogeneity of stress response patterns in the real world, the model identifies four stress response patterns: Sensitive (secure environments, high HPA reactivity enhancing adaptation); Buffered (moderate stress, low reactivity maintaining stability); Vigilant (dangerous environments, high sympathetic and HPA reactivity driving anxiety/aggression); and Unemotional (extreme stress, low reactivity across systems with high-risk behaviors) (Boyce and Ellis, 2005).

Previous studies have validated the stress adaptation patterns proposed by the ACM model from different perspectives, but results across studies have been inconsistent. Previous research measured physiological stress levels at baseline and during experimental tasks, with results preliminarily supporting the four stress response patterns of ACM. These studies also explored differences in response patterns between sexes and their relationship with early environmental stress (Del Giudice et al., 2012; Ellis et al., 2017). However, another study identified only three main stress response patterns: sensitive pattern, buffered pattern, and unemotional pattern. That study did not identify the vigilant pattern described in ACM theory (Schwartz et al., 2023). This indicates the complexity of stress adaptation patterns in the real world, requiring further validation. Additionally, previous studies have mostly focused on immediate responses of the stress system in specific situations, while beyond its manifestation in stress responses observed in immediate situational task experiments, this heterogeneous pattern of the stress system may also differ in functional dimensions such as metabolism and inter-systemic balance. Stress-related hormones such as cortisol and cortisone can be quantified in hair through diffusion from blood to hair follicle cells, providing the possibility to examine long-term adaptive functions of the SRS (Meyer and Novak, 2021; Gerritsen et al., 2019; Greff et al., 2019).

Stress responses are inherently multisystemic, involving the coordinated engagement of several biological systems, including the HPA axis, the autonomic nervous system (ANS), the hypothalamic–pituitary–gonadal (HPG) axis, and immune-related processes (Juster et al., 2010; Del Giudice et al., 2011). Rather than functioning in isolation, these systems interact and maintain a functional balance that is critical for effective stress regulation and adaptation (McEwen and Akil, 2020; Slavich, 2020). Individual differences in stress responsivity are therefore thought to reflect variation in the coordination and balance across systems, rather than the activity of any single system alone.

The HPA axis, as one of the core systems of the stress response, coordinates adrenal hormone release to maintain homeostasis (Koss and Gunnar, 2018). Cortisol and dehydroepiandrosterone (DHEA) are two key hormones secreted by the adrenal glands: cortisol suppresses inflammatory responses and promotes gluconeogenesis, while DHEA can buffer the neurotoxicity of elevated cortisol, a relationship that can translate into stress reduction and improved mental health (Gallagher and Young, 2002). Existing research shows that the cortisol/DHEA ratio (CD) provides a more comprehensive reflection of HPA axis functional status than single indicators (Ahmed et al., 2023). During puberty, DHEA levels rise significantly, while environmental changes intensify cortisol reactivity, making the CD ratio a sensitive biomarker (Foilb et al., 2011). Under stress, hormone cascade reactions are activated, leading to cortisol secretion, which is subsequently metabolized to cortisone through type 2 11-*β* hydroxysteroid dehydrogenase (11β-HSD). Cortisone can be converted back to cortisol through the action of 11β-HSD type 1. Cortisone concentration reflects the systemic metabolism of cortisol, and the interconversion between cortisol and cortisone is an important marker of glucocorticoid regulation and internal activity. Their ratio can serve as a surrogate indicator of 11β-HSD (types 1 and 2) activity (Meyer and Novak, 2021; Rippe et al., 2016; Molenaar et al., 2019; Staufenbiel et al., 2015).

Under normal conditions, the body maintains a dynamic balance of cortisol activation, metabolism, and antagonistic effects to regulate the HPA axis response to stress and maintain optimal basal cortisol levels (Gjerstad et al., 2018). Optimal CD and CC ratios are essential for adaptive stress responses, with deviations indicating maladaptation. High CD reflects cortisol dominance with potential neural damage, while low CD may impair acute stress responses (Kamin and Kertes, 2017a). Similarly, high CC indicates cortisol accumulation and disrupted regulation, whereas low CC compromises stress adaptability (Chapman et al., 2013; Koss and Gunnar, 2018).

Accumulating evidence indicates that stress adaptation is shaped by both sex and socioeconomic context, with individuals showing heterogeneous patterns of stress system functioning. Previous research suggests that sex-related differences in stress responsivity emerge across development and may reflect interactions between biological maturation and environmental demands, contributing to distinct stress adaptation patterns (Ellis et al., 2017; Kudielka and Kirschbaum, 2005; Oldehinkel and Bouma, 2011). Similarly, a growing body of research links socioeconomic disadvantage and chronic environmental stress to altered cortisol levels. Childhood socioeconomic disadvantage significantly increases the risk of mental health problems across the lifespan (Fellmeth et al., 2018; Lu et al., 2020). Socioeconomic disadvantage shapes children’s proximal environments, leading to prolonged exposure to chronic stressors. Chronic stress can induce adaptive changes in the regulation of the HPA axis, thereby affecting the stress response system; however, the direction of these associations varies across studies, with evidence for both elevated and blunted cortisol profiles under conditions of prolonged stress exposure (Merz et al., 2024; Simon et al., 2021; Tian et al., 2021). Together, these inconsistent findings highlight substantial heterogeneity in HPA axis functioning and underscore the need for theory-driven approaches that explicitly account for sex- and context-related variation in stress system calibration.

In summary, although previous research has validated the ACM model, these studies were based on immediate responses under experimental conditions. Moreover, the adaptive adjustment of the HPA axis includes not only baseline levels and situational adaptability but also variability in broader aspects of stress system functioning, including metabolic regulation and physiological balance among multiple systems (Ellis et al., 2017). Previous markers in saliva, urine, and blood inadequately capture the long-term functional state of the HPA axis. Hair HPA axis stress markers, with their stability and reliable retrospective nature, have been extensively applied in stress-related studies of HPA axis function (Zerroug et al., 2025; Stalder et al., 2017). Additionally, the CD ratio and CC ratio combined with cortisol indicators may improve the identification of heterogeneous patterns of HPA axis function (Zerroug et al., 2025). Therefore, this study adopts an ACM-informed perspective to explore whether individual differences previously observed in acute stress reactivity may also be reflected in biomarkers indexing recent chronic stress-related physiological functioning. Furthermore, it is hypothesized that the levels of perceived stress and depressive symptoms will systematically differ across these patterns and distribution of heterogeneous stress adaptation patterns will vary by sex and socioeconomic disadvantage.

## Methods

This study was approved by the Ethics Committee of the Affiliated Hospital of Xuzhou Medical University (XYFY2024-KL586-01). The study was conducted between July and September 2024. Subjects were recruited from middle schools, and each participant and their guardian voluntarily signed a paper informed consent form. Eligible subjects completed paper questionnaire assessments and provided 1 cm hair samples from the posterior occipital region near the scalp under the guidance of on-site researchers. The study assumes an average hair growth rate of approximately 1 centimeter per month, thus a 1-centimeter sample likely represents the past month’s accumulation (Meyer and Novak, 2021). Inclusion criteria were as follows: no hair perming or dyeing within the past month; no major stressful events (earthquakes, floods, deaths of relatives or friends, etc.) experienced in the past 6 months; no hormone medications taken in the past month. Exclusion criteria: presence of major mental or physical illness; insufficient hair (less than 1 cm) in the posterior occipital region.

### Assessment tools

Demographic questionnaire: This questionnaire was compiled based on the purpose of the study and included age, sex, grade, and other sociodemographic data to assess the specific problems of disadvantaged adolescents (such as parents’ marital status, annual household income, parents’ length of work outside the home, home environment [house size, quality of decoration, geographical location, etc.]). Hair washing frequency was self-reported as the average number of days between hair washes and was included as a covariate because it may influence hair cortisol concentrations (Qi et al., 2024).

Following previous studies(Wang et al., 2025; Li et al., 2025; Cao, 2016), children from disadvantaged backgrounds were classified based on the sociodemographic background: ① single-parent families; ② left-behind children (both parents or one parent working outside for more than 6 months); ③ migrant children (who had been studying outside of their registered residence for more than 6 months); ④ children with low socioeconomic status were evaluated based on the following items in the demographic questionnaire: annual income of households (lower than the local average level was recorded as 1, higher than the average level was recorded as 0); the educational level of the parents (if the educational level of fathers and/or mothers was only primary school and below it was recorded as 1, and educational level above primary school was recorded as 0); the ownership of household appliances (“computers, TVs, air conditioners, refrigerators and washing machines”, if there were 0–1 kinds, it was recorded as 1, and if there was more than 1 kind, it was recorded as 0); and subjective home environment was assessed by a self-reported item reflecting adolescents’ overall perception of their home environment (including housing size, quality, household facilities, and geographic location). Responses were categorized as poor, general, or good, with poor coded as 1 and general/good coded as 0. The scores of the four conditions were added together and if the total score was greater than or equal to 3, the children were considered to be disadvantaged.

The Children’s Depression Inventory (CDI) (Kovacs, 1985) was employed to measure depression. The CDI consists of 27 self-report items. Each item on the scale is scored as 0, 1, or 2, with higher scores indicating more severe depressive symptoms. A total score greater than 19 is considered indicative of depression. The reliability and validity of the Chinese version have been validated in several studies (Wu et al., 2010; Hong Xin et al., 2012; Tian Yiyao et al., 2023).

Perceived Stress Scale-10 (PSS-10) was used to assess perceived stress of adolescents over the past month. Its scores range from 0 to 40, which derive from 10 items, and are positively correlated with stress levels (Cohen et al., 1983). The PSS-10 has proved to be more applicable than the PSS-14 for measuring the perceived stress in a large community-based population in China (Huang et al., 2020).

Simultaneous determination of the five steroids in hair: The three steroids were simultaneously determined by high performance liquid chromatography tandem mass spectrometer (LC–MS/MS). Firstly, three steroids are extracted and purified form hair samples by washing, incubation, solid phase extraction and redissolution. Then, they are quantified by the LC–MS/MS system consisting of an Agilent 1,200 HPLC system and AB Sciex 3,200 QTRAP tandem mass spectrometer with atmospheric pressure chemical ionization source in positive mode. The details of the analysis method were reported in previous studies (Chen et al., 2019; Qi et al., 2024).

The investigation procedure of this study is as follows: This research employed a convenience sampling method, and the sample was drawn from a secondary school. All participants voluntarily signed up for the study after fully understanding its purpose and content, and participants who completed the questionnaires were reimbursed with a cash amount of CNY50. Students who agreed to participate would first be asked a series of standardized questions about whether they had experienced any significant traumatic events in the past 6 months, as well as if they had taken any medications or suffered from other major illnesses in the past month. Individuals who met all the inclusion criteria would further complete a paper questionnaire and provide hair samples. All participating individuals were typically developing.

### Statistical analysis

At the outset, descriptive statistics for demographic characteristics of the sample were computed. Continuous variables are presented as the means and standard deviation. Categorical variables are presented as counts and percentages. The Shapiro–Wilk test (swilk) was used to assess the normality of data distribution.

Latent Profile Analysis (LPA) is a statistical method designed to explore the structural relationships between observed variables by identifying latent categories within the data. It primarily fits models using maximum likelihood estimation to reveal different profiles of individuals based on specific characteristics. A series including Akaike information criterion (AIC), sample size-adjusted Bayesian information criterion (saBIC), entropy and bootstrapped likelihood ratio test (BLRT) were chosen to identify the optimal solution of LPA analysis. More specifically, the lowest possible scores of AIC, BIC, and saBIC values indicate better fit (Nylund et al., 2007). Higher entropy provides information about the accuracy of the resulting latent profiles. The fact that the Lo–Mendell–Rubin Likelihood Ratio Test (LMR) and BRLT values are significant indicates a good fit (Tein et al., 2013). When determining the best category, it is often necessary to analyze multiple indicators comprehensively, while also placing significant emphasis on theoretical interpretability (Yin Kui and Jun, 2020). The percentage of individuals in a single class should be at least 5% of the total population to ensure the robustness of the results (Yin Kui and Jun, 2020).

RStudio for macOS (version 2022.02.3) was used to conduct the LPA analysis. LPA analyses were performed using the R package “tidyLPA” (version 1.1.0) (Rosenberg et al., 2018). Data analysis and graph generation were completed using GraphPad Prism10 (GraphPad Software, San Diego, California, USA). ANOVA tests were used to compare group differences. The level of significance was set to 0.05 (two-tailed).

## Results

A total of 142 participants completed the questionnaires, but 18 had to be excluded due to insufficient hair samples for analyses. This study ultimately included 124 complete samples for testing and data analysis. More than half of the sample was female, with children from disadvantaged backgrounds accounting for over 30%. Detailed demographic information is shown in Table 1. The correlation matrix between variables is shown in Table 2.

**Table 1:** Basic demographics of the study sample.

Variables	N/Mean/Median	%/SD/IQR	Value range (min-max)
Age (mean/SD)	15.54	3.08	12–18
Sex (N/%)			
Male	52	41.94	
Female	72	58.06	
Status (N/%)			
Non-disadvantaged	81	65.32	
Disadvantaged	43	34.68	
Cortisol(pg/mg)	5.55	3.35–8.1	1–13.88
Cortisol/Cortisone	0.78	0.43–1.24	0.10–5.24
Cortisol/DHEA	0.38	0.19–0.77	0.05–2.43
Hair washing frequency (days between washes)	1.87	0.62	1–3
CPSS	17.31	5.62	3–35
CDI	10.71	7.23	0–39

SD, standard deviation; IQR: Interquartile Range; CPSS: Chinese Perceived Stress Scale; CDI: Children’s Depression Inventory; DHEA: Dehydroepiandrosterone.

**Table 2:** The correlation matrix between variables.

Variables	Cortisol	Cortisol/Cortisone	Cortisol/DHEA	CDI	CPSS
Cortisol	1.00				
Cortisol/Cortisone	0.75**	1.00			
Cortisol/DHEA	0.56**	0.42**	1.00		
CDI	0.06	0.11	−0.05	1.00	
CPSS	0.22*	0.19*	0.05	0.58**	1.00

CPSS: Chinese Perceived Stress Scale; CDI: Children’s Depression Inventory; DHEA: Dehydroepiandrosterone. *p < 0.05; **p < 0.01.

The model fits of LPA are shown in Table 3. The AIC and sample size-adjusted BIC did not reach a minimum, and the BLRT was significant for each successive model. The entropy, as the index of classification accuracy of model fit, decreased with increasing model complexity. The model fit index does not explicitly point to model selection schemes. In the model selection, the interpretability and stability of the model should also be considered. Based on the consideration above, a four-profile solution was accepted in our study.

**Table 3:** Model fit statistics for the 1- to 5-profile solutions.

**Classes**	**AIC**	**saBIC**	**Entropy**	**Prob_min**	**Prob_max**	**N_min**	**BLRT_var**	**BLRT_p**
1	1,065	1,082	1	1	1	1		
2	827	864	0.91	0.98	0.98	0.40	252	0.009
3	754	810	0.93	0.89	0.98	0.07	87.1	0.009
**4**	**728**	**804**	**0.89**	**0.90**	**0.99**	**0.15**	**39.9**	**0.009**
5	713	809	0.89	0.83	0.97	0.03	28.8	0.020

AIC = Akaike Information Criterion; saBIC = sample-size adjusted Bayesian Information Criterion; Prob_min = minimum posterior class membership probability; Prob_max = maximum posterior class membership probability; N_min = minimum class size; BLRT_var = bootstrap likelihood ratio test variance; BLRT_p = bootstrap likelihood ratio test *p-*value. Bold typeface indicates the best model.

**Figure 1:**
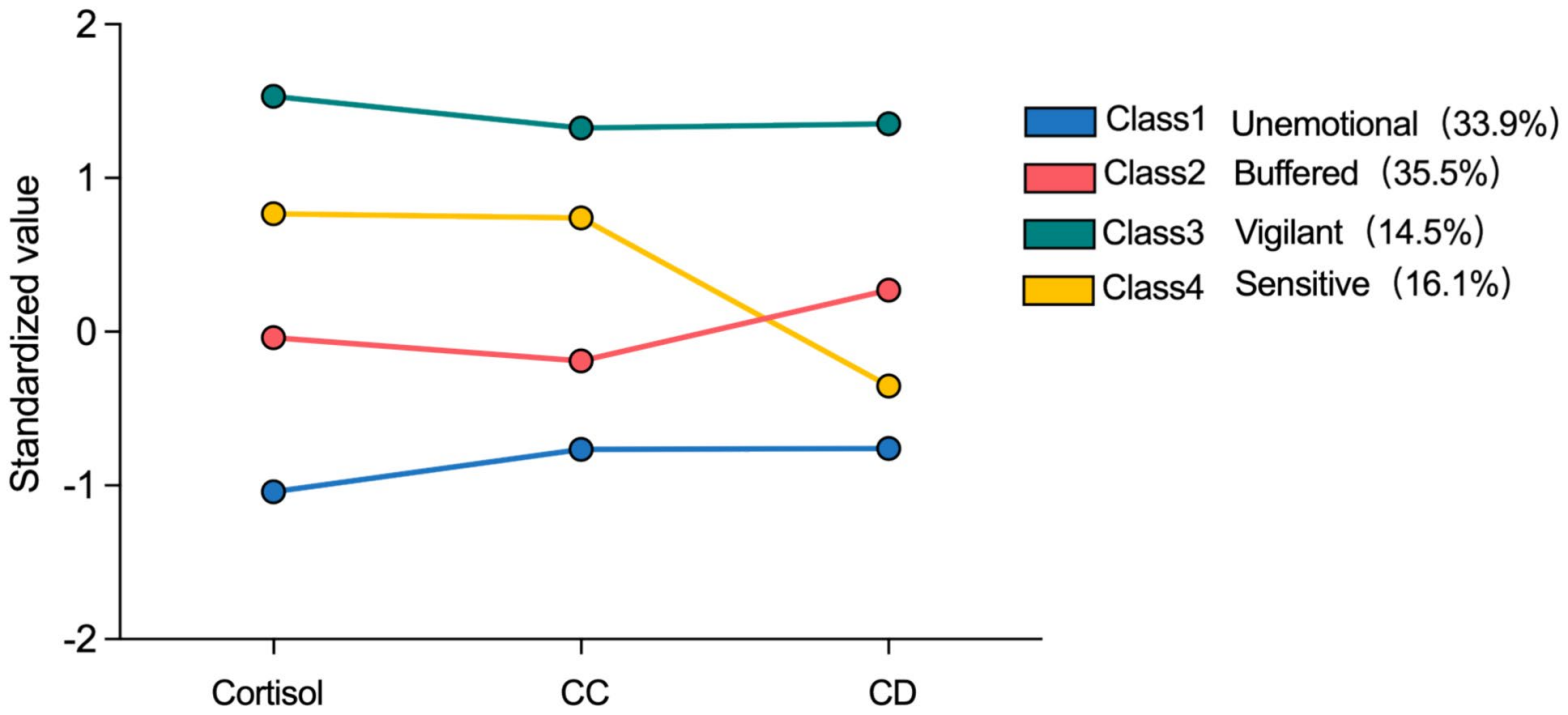
The four-profile model based on the performance of HPA function. HPA: Hypothalamic–Pituitary–Adrenal axis.

The characteristics of each group are shown in Figure 1. Group 2 had the highest proportion at 35.5%, while Group 3 had the lowest at 14.5%. Analysis of variance results showed no statistically significant differences between groups in age and sex (*p* = 0.65 for age differences between groups; *p* = 0.60 for sex differences between groups).

**Figure 2:**
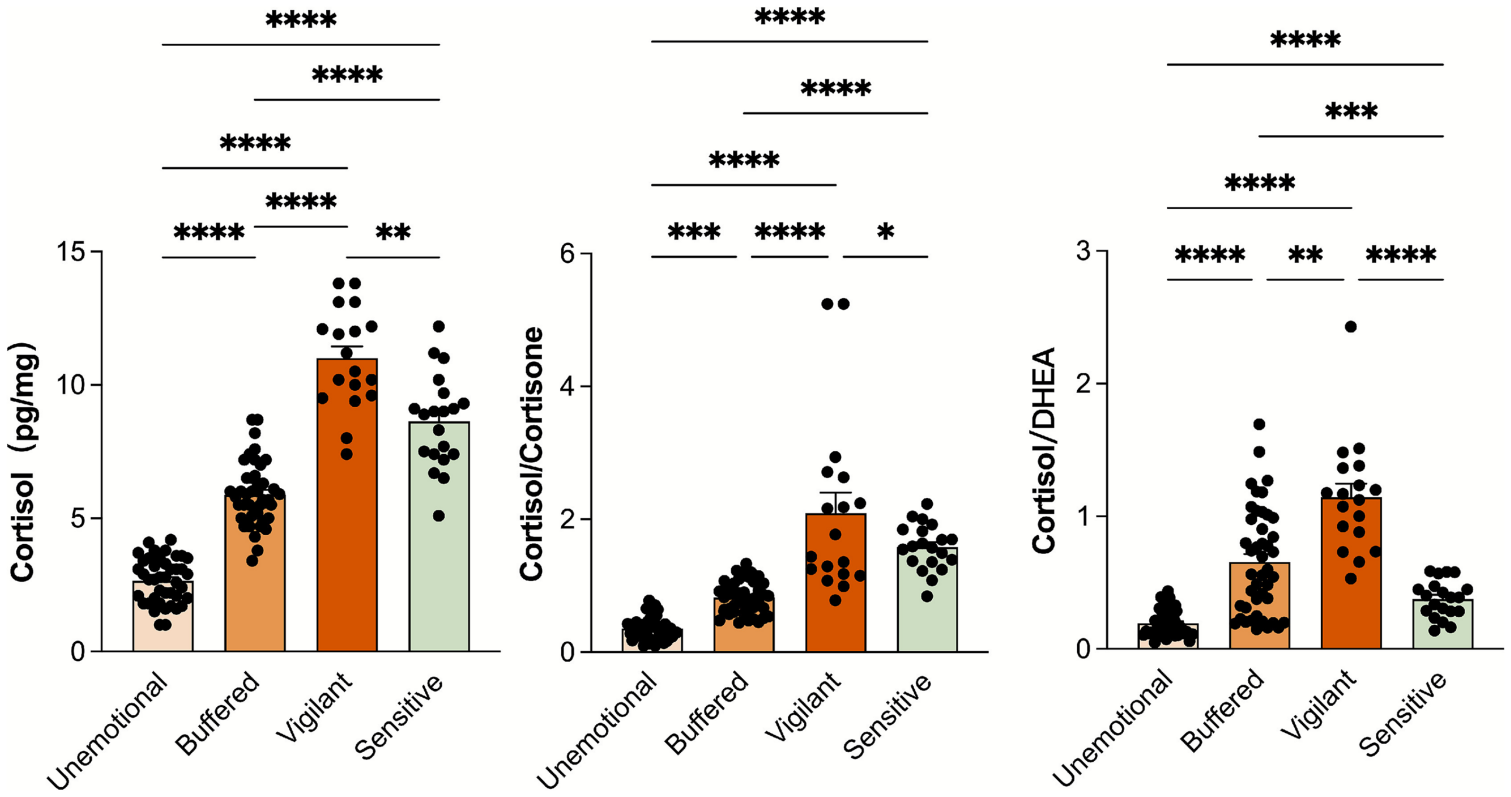
Differences in Cortisol, CD values, and CC values among different groups. DHEA: Dehydroepiandrosterone; cortisol/DHEA ratio (CD), and cortisol/cortisone ratio (CC). *p < 0.05; **p < 0.01; *** *p* < 0.001; ****p < 0.0001.

Figure 2 displays the differences in the three HPA axis stress parameters between groups. After controlling for age, sex and perceived stress level, the results of the inter-group differences indicate that the differences among the groups in the three indicators are statistically significant, demonstrating the effectiveness of heterogeneous grouping. Individuals in Class 1 exhibit stress physiological characteristics of low cortisol, low CC, and low CD. This category of individuals has a generally weak ability to respond to stress and show low responsiveness and adaptability to environmental stimuli, corresponding to the unemotional type in ACM. Class 2 include relatively low cortisol, relatively low CC, and relatively high CD. Individuals in this class have a moderate stress response capability, accompanied by a high CD value. In the real world, their stress adaptation still primarily relies on cortisol, which helps individuals adapt better, corresponding to the buffered type in ACM. Class 3 individuals display stress physiological characteristics of high cortisol, high CC, and high CD. This type shows elevated cortisol levels, which includes high secretion of cortisol and low activity of 11β-HSD that leads to cortisol accumulation, indicating strong perception and responsiveness to environmental stress, with a high physiological activation to respond to potential threats. Additionally, a high CD ratio also represents a high risk for HPA dysfunction, corresponding to the vigilant type in ACM. Finally, Class 4 exhibits relatively high cortisol, relatively high CC, and relatively low CD. This group maintains high sensitivity and responsiveness under stress, while the low CD value exacerbates their maladaptation, corresponding to the sensitive type in ACM.

**Figure 3:**
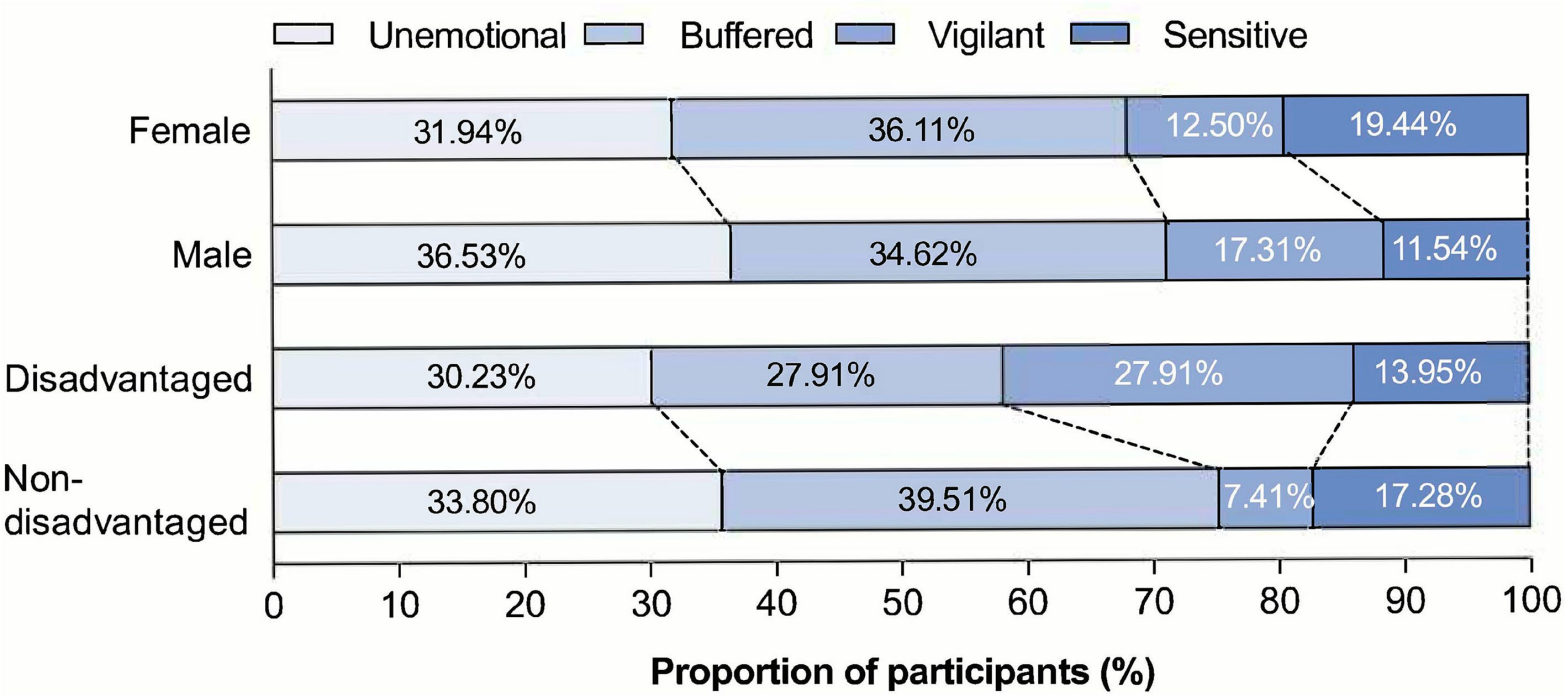
HPA axis adaptation categories across sexes and disadvantaged conditions. HPA: Hypothalamic–Pituitary–Adrenal axis.

Figure 3 illustrates differences in stress adaptation patterns across different sexes and disadvantaged groups. The results showed that although there were differences between male and female across the stress subtypes, none of them reached statistical significance. Children from disadvantaged backgrounds, compared to non-disadvantaged children, are characterized by a significant difference in the Vigilant type (*z* = 2.76, *p* = 0.006).

**Figure 4:**
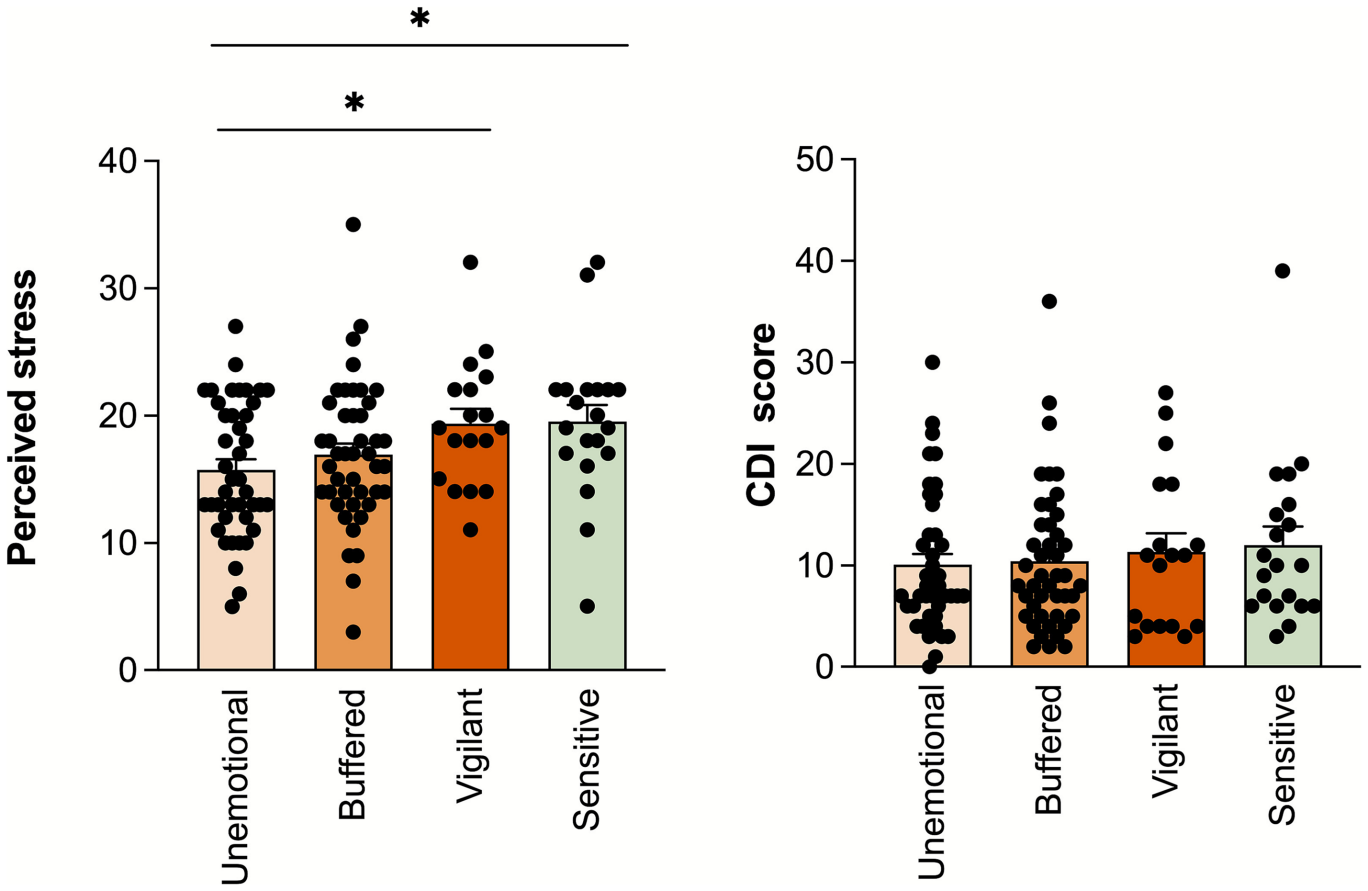
Differences in perceived stress and CDI scores among different groups. CDI: Children’s Depression Inventory.

Figure 4 shows differences in perceived stress and depression symptom scores among different stress adaptation patterns. After controlling for age and sex, results showed that, relative to the Unemotional group (Group 1), the Vigilant group (Group 3; *t* = 2.46, *p* = 0.017) and the Sensitive group (Group 4; *t* = 2.28, *p* = 0.026) reported higher perceived stress levels. After controlling for age, sex and subjective perceived stress levels, there were no statistically significant differences in depression symptom scores between groups (*p* > 0.05).

## Discussion

This study is the first to systematically explore heterogeneous patterns of stress system adaptability in real-world conditions by combining cortisol, CD ratio, and CC ratio in hair as multidimensional functional markers of the HPA axis. Previous studies have mostly adopted experimental scenarios, using individuals’ stress responses in stress-inducing tasks—such as the cortisol awakening response (CAR), heart rate variability, and similar indicators—to validate heterogeneous patterns of stress (Del Giudice et al., 2011; Ellis et al., 2017). Compared with momentary indicators, hair cortisol and its related ratios (cortisol/DHEA, cortisol/cortisone) reflect cumulative glucocorticoid secretion, negative feedback, and metabolic balance over an extended period (Kamin and Kertes, 2017b; Qi et al., 2024). They therefore better indicate recent chronic stress–related patterns of HPA axis activity. This study supplements research approaches based on CAR, aiming to provide a multidimensional perspective on the heterogeneity of chronic HPA axis functional characteristics.

The results revealed four distinct stress adaptation patterns, indicating significant differences in adolescents’ physiological responses to stress, consistent with previous research findings (Del Giudice et al., 2012; Ellis et al., 2017). It is suggested that early adverse experiences or environmental exposures can lead individuals to be stress-sensitive or stress-inoculated; the former results in increased cortisol production, while the latter leads to elevated levels of DHEA, which also explains the heterogeneity in stress response patterns (Ozbay et al., 2008; Saczawa et al., 2013; Bendezú et al., 2021). Notably, significantly elevated and decreased CD ratios are associated with depression and aggression, respectively (Angold, 2003; Pajer et al., 2006; Saczawa et al., 2013). The unemotional type exhibits low cortisol secretion and high cortisol metabolism, resulting in a lack of necessary regulatory ability in stressful situations, thus showing weaker stress response capabilities in their environment. At the same time, a low CD ratio indicates that, despite low cortisol, the level of DHEA does not effectively compensate, likely due to the frequent activation of the HPA axis leading to lower baseline cortisol levels and a compensatory prioritization of DHEA production over cortisol in the face of long-term stress (Lee et al., 2024). The vigilant type, characterized by high cortisol secretion and low metabolism, shows heightened physiological activation to respond to potential threats, with strong perception and response capabilities to environmental stressors. A high CD ratio reflects greater susceptibility to HPA axis dysfunction (Qiao et al., 2017), indicating that while they can quickly perceive stress, the levels of DHEA are insufficient to offset the negative impacts caused by it.

The buffered type maintains moderate cortisol levels, with a lower CC ratio indicating that their cortisone levels are not excessive, demonstrating strong psychological resilience to better cope with external stress (Lau et al., 2021). A higher CD ratio suggests that, despite lower cortisol secretion levels, they primarily rely on cortisol for stress coping, which helps them better adapt to environmental pressures in the real world (Saczawa et al., 2013). The sensitive type has relatively high overall cortisol levels, indicating they are more sensitive to stress. A higher CC ratio suggests low activity of 11β-HSD, contributing to cortisol accumulation. In the short term, this may help them respond effectively to stress; however, a lower CD ratio indicates that despite higher cortisol secretion, the supportive role of DHEA is relatively insufficient, which may lead them to primarily rely on DHEA secretion when coping with stress, thereby reducing their adaptability (Ozbay et al., 2008; Saczawa et al., 2013).

These results further support the theoretical models of ACM and contribute to a deeper understanding of stress response pattern mechanisms and the design of personalized interventions. However, a previous study identified only three main stress response patterns: sensitive pattern, buffered pattern, and unemotional pattern, without identifying the vigilant pattern (Schwartz et al., 2023). This indicates the complexity of individual SRS in the real world, and future studies should further enrich model validation.

This study found no significant differences in stress patterns between males and females, which is inconsistent with previous research (Del Giudice et al., 2012). Del Giudice et al. (2012) found in their study that in high-stress environments, male children were more likely to exhibit the unemotional pattern, while female children were more likely to exhibit the Sensitive pattern. ACM theory points out that the male HPA axis is more sensitive to achievement stress, exhibiting emotional blunting and risk preference, achieving competitive risk-taking by reducing reactivity; females are more sensitive to social rejection, developing care-friendship patterns, and evolving into high reactivity-low anxiety sensitive phenotypes in safe environments, promoting parental investment (Ellis and Boyce, 2008; Del Giudice et al., 2011; Ellis and Del Giudice, 2019; Ellis et al., 2017). Most existing validation studies of the ACM model have focused on Western adolescent populations (Ellis et al., 2017; Del Giudice et al., 2012), and these differences in results may be due to sociocultural and methodological factors (such as stress measurement indicators and ways of operationalizing stress) (Ellis and Del Giudice, 2019). Future research should test the applicability and robustness of ACM in a broader population to enhance its generalizability and ecological validity.

Children from disadvantaged backgrounds, compared to non-disadvantaged children, were characterized by the Vigilant type as a significant feature difference, indicating their focus on threats and high trait anxiety in high-stress environments (Del Giudice et al., 2012; Ellis et al., 2017). The vigilant response pattern is an evolutionarily formed conditional adaptive mechanism that addresses environmental threats through early maturation, low somatic investment, and high-risk behaviors (such as impulsive aggression). Children from disadvantaged backgrounds optimize survival in high-stress environments through physiological-behavioral integration, achieving environmental adaptation by enhancing threat monitoring capabilities, but at the cost of long-term health risks (such as cardiovascular disease) and psychological pathology tendencies (Ellis et al., 2017; Del Giudice et al., 2011; Ellis and Del Giudice, 2019).

In this study, the buffered type had the highest proportion (35.5%), consistent with previous research (Ellis et al., 2017), indicating that for most children, exposure between two extreme environments will downregulate sensitivity, thereby buffering chronic stress encountered in a real world that is neither highly threatening nor absolutely safe (Boyce and Ellis, 2005). This study did not find differences in depression scores between different stress response patterns, inconsistent with previous research findings (Del Giudice et al., 2012). This may be because previous research found that stress response adaptation patterns may be more related to externalization problems (Lee et al., 2024; Salis et al., 2016). More importantly, previous research results indicate that the impact of perceived stress, stress response, and outcomes such as depression may be moderated by factors such as cognition and coping styles (Lehrer et al., 2020). Individuals with higher psychological resilience may have more stable cortisol responses under high stress (Lehrer et al., 2020). In other words, when individuals cope with stressful events in the real world, their adaptation process is influenced not only by the physiological stress system but also by coping styles. High-response types (Vigilant, Sensitive) may maintain reasonable stress levels through the regulation of coping styles to promote adaptation; similarly, low-response types (Unemotional and Buffered) may exhibit high physiological stress levels during acute stress through the regulation of coping styles, thereby maintaining normalization of individual emotional and behavioral adaptation (Del Giudice et al., 2011). The mechanisms of these individual differences (such as coping strategies and resilience) in the relationship between physiological stress responses and emotional behavioral outcomes need to be further explored in future research.

## Conclusion

This study is the first to validate the stress adaptation patterns proposed by the ACM model from multidimensional functional indicators of HPA axis functional adaptation perspective, and further found that early disadvantaged environments may foster a vigilant stress adaptation pattern. The vigilant and Sensitive types were more likely to perceive stress, while physiological stress patterns had no effect on depression. The results provide reference for further mechanism analysis and intervention.

## Limitations

This study classified disadvantage based only on sociodemographic data, while early stress environments are more complex. Future research should fully distinguish between trauma and disadvantaged experiences to better identify the impact of early environments on SRS (Hosseini-Kamkar et al., 2021). Additionally, although this study used multi-indicators to evaluate the adaptive function of the HPA axis, stress responses involve more complex mechanisms in the body. Future research should further integrate different physiological systems (sympathetic nervous system, parasympathetic nervous system, and HPA axis) to assess physiological adaptation patterns of stress (Glier et al., 2022). Furthermore, previous studies have mostly assessed the stress system based on baseline (static) stress assessment and laboratory functional tasks (dynamic). Although this study assessed the multidimensional functional level of the HPA axis based on hair matrix, assessing stress responses in more natural environments, for example, using ecological momentary assessment methods, would provide more authentic stress response data. Finally, given the small sample size and the statistical ambiguity, caution should be exercised when extending the four-category findings to a broader population of adolescents.

## Data Availability

The raw data supporting the conclusions of this article will be made available by the authors, without undue reservation.
